# Shapiro Syndrome: A Case Report

**DOI:** 10.7759/cureus.97326

**Published:** 2025-11-20

**Authors:** Manya Motsara

**Affiliations:** 1 General Medicine, King's College Hospital NHS Foundation Trust, Kent, GBR

**Keywords:** agenesis of the corpus callosum, elderly female, episodic hypothermia, general internal medicine, generalized hyperhidrosis, neurology, rare disease or condition, shapiro syndrome

## Abstract

Shapiro syndrome is a rare dysautonomic disorder defined by the triad of spontaneous recurrent hypothermia, hyperhidrosis, and agenesis of the corpus callosum. Recognition in older adults is uncommon and often delayed, as episodes can mimic infection or endocrine disease.

We report an 81-year-old woman with established Shapiro syndrome who presented with recurrent admissions for profound hypothermia (31.3-33.6 °C) accompanied by delirium and sinus bradycardia. Previous MRI demonstrated complete agenesis of the corpus callosum with colpocephaly and hippocampal hypoplasia. Episodes were managed with active rewarming, hydration, and adherence to a neurology plan emphasizing conservative temperature control and avoidance of antipyretics during hypothermia.

For prophylaxis, she received clonidine 25 mg (three tablets in the morning, two at midday, and three at night) and lamotrigine 50 mg twice daily for background epilepsy. With this protocol, episode severity and duration decreased. This case highlights classic Shapiro syndrome persisting into advanced age. In elderly patients with unexplained recurrent hypothermia and sweating, especially with documented agenesis of the corpus callosum, early recognition and a structured conservative plan can reduce hospitalizations and iatrogenic interventions.

## Introduction

Shapiro syndrome, first described in 1969 [1], is characterized by episodic spontaneous hypothermia with hyperhidrosis and, in its classic form, agenesis of the corpus callosum (ACC). ACC refers to the congenital absence of the major white-matter tract connecting the cerebral hemispheres. The syndrome arises from hypothalamic dysregulation of thermoregulation and autonomic tone, leading to recurrent temperature dyscontrol [2].

Although most reports involve children or younger adults, a subset of patients present or persist into later life [3]. In older adults, attacks are frequently mistaken for sepsis or endocrine emergencies, leading to repeated investigations and admissions.

Episodes may be mistaken for infection, hypothyroidism, or adrenal crises, resulting in delayed diagnosis. Although most reported cases occur in children or younger adults, persistence into advanced age is exceptionally rare. The pathophysiology likely involves serotonergic, dopaminergic, and noradrenergic imbalance within the hypothalamus.

We present an octogenarian with classic Shapiro syndrome (complete ACC) and recurrent hypothermic episodes complicated by delirium and bradycardia. The case emphasizes practical management and care-home protocols.

## Case presentation

An 81-year-old female resident of a care home with a background history of Shapiro syndrome, epilepsy (head-injury related), multi-infarct dementia, hypertension, acute coronary syndrome, osteoporosis, and ocular motility disorders presented with lethargy, reduced oral intake, and confusion. Over the preceding week, she had multiple hypothermic episodes with profuse sweating and intermittent chest discomfort. Recorded core temperatures were 31.3-33.6 °C. This was her third presentation in seven days for similar symptoms.

On examination, her temperature was 31.8 °C, blood pressure 130/68 mm Hg, pulse 48 bpm (sinus bradycardia), respiratory rate 16/min, and oxygen saturation 98% on room air. She was diaphoretic and pale with hypoactive delirium (GCS 14/15) [4]. Chest, cardiac, and abdominal examinations were otherwise unremarkable; there was no meningism or focal neurological deficit aside from psychomotor slowing. ECG revealed sinus bradycardia with lateral T-wave inversion (previously attributed to hypothermia); troponin T was 6 ng/L without dynamic change. Laboratory tests showed low inflammatory markers and mild hypernatremia consistent with dehydration, which corrected with IV 5% glucose. Renal, hepatic, thyroid, and cortisol panels were otherwise normal (Table 1). Blood and urine cultures were negative.

**Table 1:** Laboratory results on admission showing mild hypernatremia due to dehydration during hypothermia, with otherwise normal hematologic, renal, and endocrine parameters. These findings excluded infection or endocrinopathy as causes of temperature dysregulation.

Parameter	Patient Value	Result	Normal Range	Comment
Core temperature	31.3–33.6 °C	Marked hypothermia	36.0–37.5 °C	Hypothermic episode
Sodium	146 mmol/L	Mild hypernatremia	135–145 mmol/L	Due to dehydration
Potassium	3.6 mmol/L	Normal	3.5–5.0 mmol/L	No abnormality
Creatinine	1.0 mg/dL	Normal	0.5–1.2 mg/dL	No abnormality
Blood urea nitrogen	10 mg/dL	Normal	7–20 mg/dL	No abnormality
Glucose	85 mg/dL	Normal	70–110 mg/dL	No abnormality
Cortisol	15 µg/dL	Normal	5–25 µg/dL	No abnormality
Thyroid profile (TSH)	2 µIU/mL	Normal	0.4–4.0 µIU/mL	No abnormality
C-reactive protein (CRP)	<5 mg/L	Normal	< 5 mg/L	No inflammation
Troponin T	6 ng/L	Normal	< 14 ng/L	No acute ischemia
White blood cells	8 × 10⁹/L	Normal	4.0–11.0 × 10⁹/L	No infection
Hemoglobin	11 g/dL	Mildly reduced	12–16 g/dL	Mild anaemia
Platelets	285 × 10⁹/L	Normal	150–400 × 10⁹/L	No abnormality

**Figure 1:**
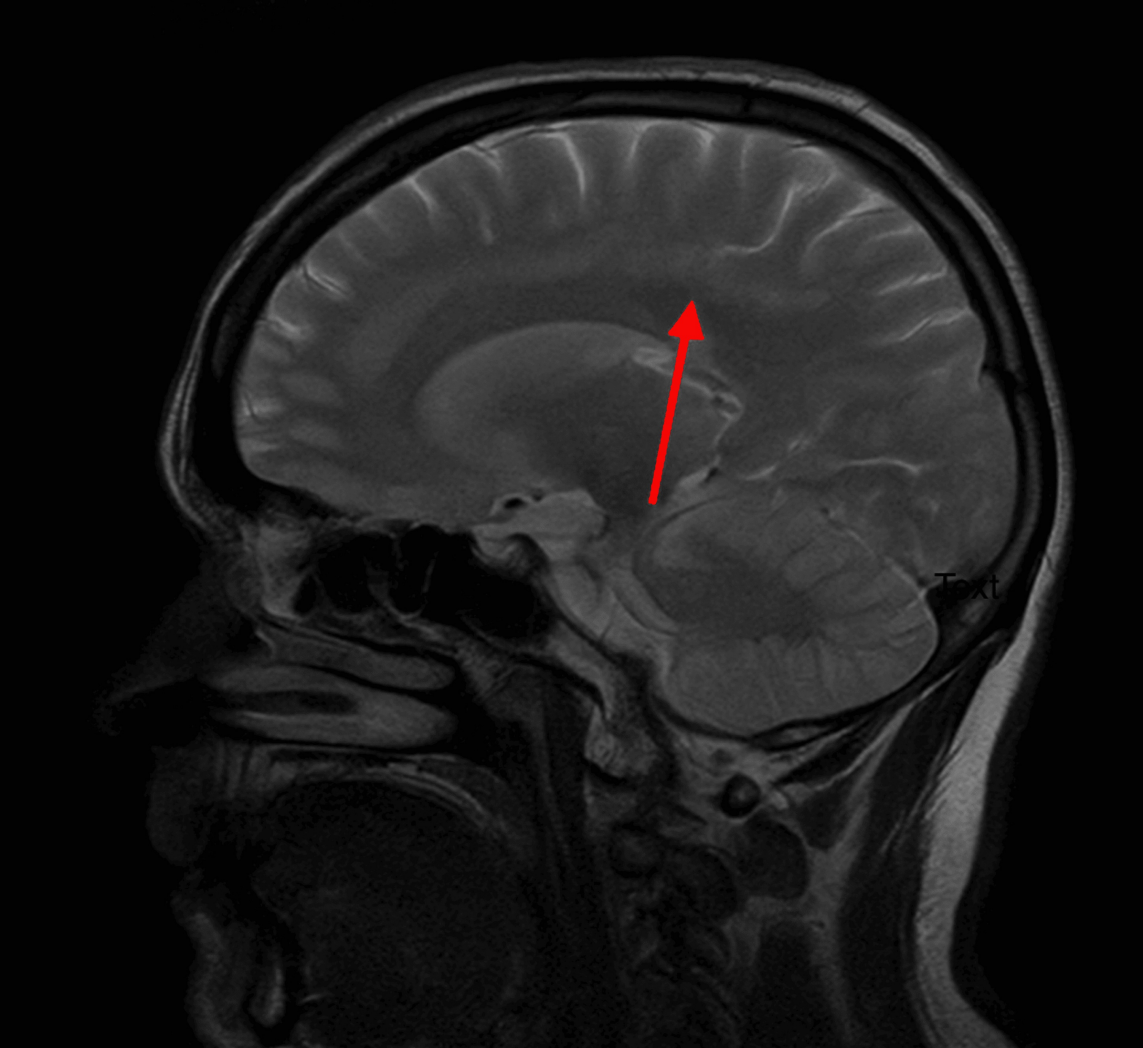
Sagittal T1-weighted MRI brain demonstrating complete agenesis of the corpus callosum with absence of callosal fibers and a high-riding third ventricle.

**Figure 2:**
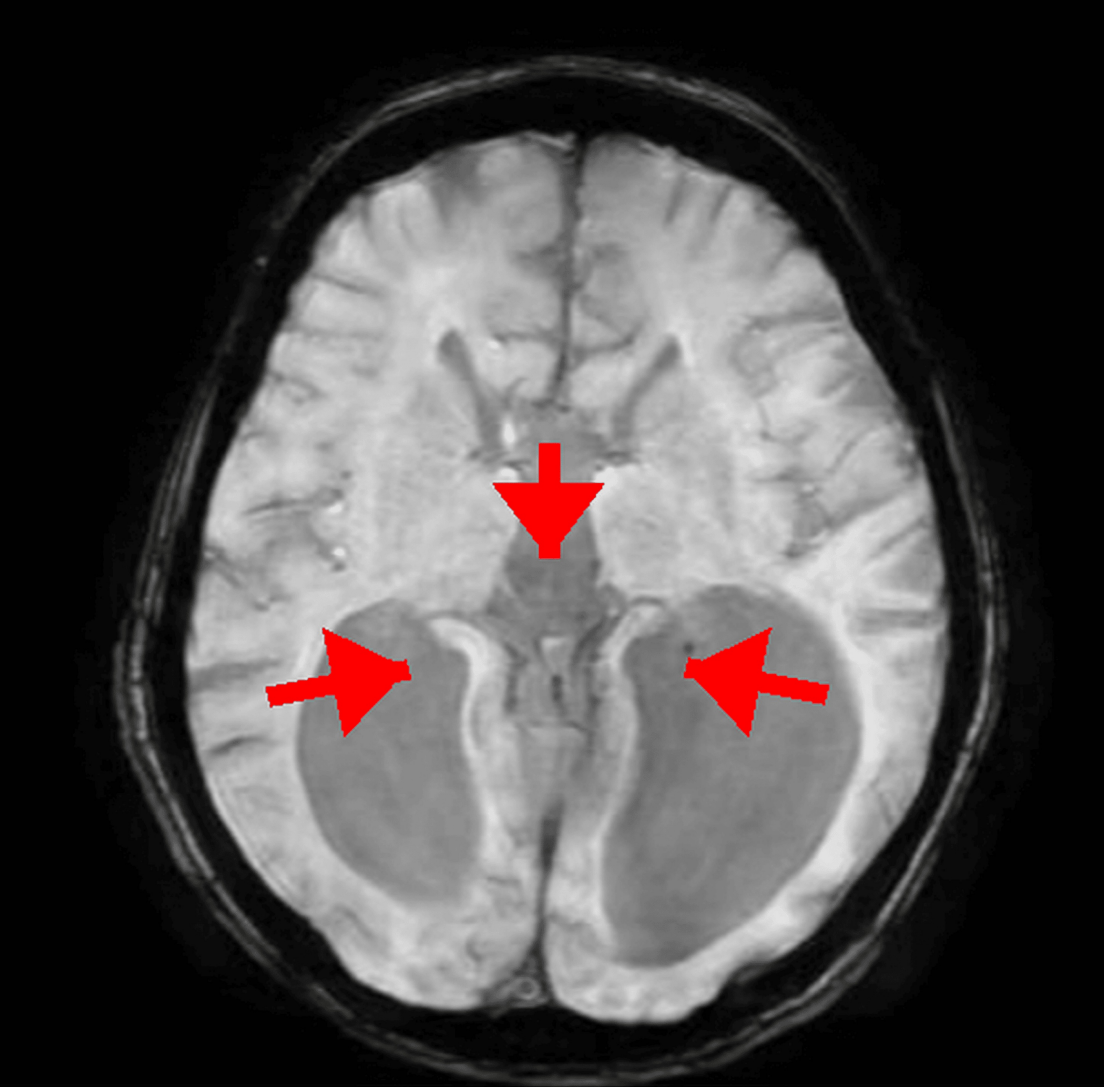
Axial T1-weighted MRI brain illustrating radial arrangement of gyri and enlarged occipital horns, characteristic of colpocephaly in callosal agenesis.

MRI head with contrast was compared with scans from June 2020 and July 2022, revealing complete agenesis of the corpus callosum with radially arranged gyri pointing toward the third ventricle, widely separated parallel lateral ventricles with trident-shaped anterior horns, Probst bundles, absent cingulate gyrus, high-riding third ventricle, colpocephaly, and hippocampal hypoplasia (Figures 1, 2). Mature, stable damage was noted in the left temporal lobe, right inferior frontal lobe, and left cerebellum. No acute infarction or hemorrhage was seen.

Neurology assessment attributed recurrent admissions to Shapiro syndrome in the setting of dementia and epilepsy. Conservative management with medical optimization was advised [3]. This included maintaining the core temperature, IV fluids, and warming when hypothermic, avoiding antipyretics during hypothermic episodes (paracetamol only if >36 °C), and hospital admission if the temperature was ≤32-33 °C for more than two to three days or if delirium developed.

Medication was optimized by increasing clonidine 25 mg in a 3-2-3 TDS regimen and initiating lamotrigine 50 mg BD while discontinuing phenytoin due to toxicity [4].

During admission, she received active rewarming (Bair Hugger), warmed IV fluids, electrolyte correction, and continuous cardiac monitoring. Following neurology guidance, ambient temperature was optimized. Temperature rose to 35.5 °C within 24 hours and normalized over 48 hours; cognition improved with rewarming. No infection was identified. The patient was discharged with reinforced care-home protocols, including daily temperature checks, early fluids and blankets, medication adherence, and readmission criteria.

Following discharge, care-home protocols were reinforced, including daily temperature monitoring, hydration, medication adherence, and readmission for persistent hypothermia (≤32 °C) or delirium.

## Discussion

Shapiro syndrome is extremely rare; most reported cases are paediatric [2,3,5]. Adult and elderly presentations are increasingly recognized but remain underdiagnosed. The classic triad was fully met in this patient: recurrent spontaneous hypothermia, hyperhidrosis, and MRI-confirmed complete ACC.

The disorder reflects hypothalamic thermoregulatory dysfunction with autonomic imbalance, including simultaneous heat loss through vasodilation and sweating and impaired thermogenesis, possibly worsened by interhemispheric disconnection due to ACC [3]. Neurochemical hypotheses involve serotonergic, noradrenergic, and dopaminergic pathways, explaining benefits from agents such as cyproheptadine, clonidine, and bromocriptine in previous reports [2,6]. Lamotrigine may stabilize hypothalamic excitability in patients with epilepsy [2].

Hypothermia can induce bradyarrhythmias and T-wave inversion with minor troponin elevations, which typically normalize on rewarming [7].

These findings align with previous reports showing improved autonomic stability through α-adrenergic modulation and neuronal membrane stabilization. In elderly patients, conservative management, focusing on environmental control, hydration, and avoidance of unnecessary interventions, remains the safest and most effective approach.

There is no universally effective therapy. In frail elderly patients, conservative measures remain safest, and management should be individualized. In this case, therapy focused on environmental temperature control, routine surveillance, and admission only if the temperature was ≤32-33 °C for more than two days or if delirium occurred. Clonidine aided autonomic stabilization, lamotrigine addressed epilepsy and thermoregulation, and antipyretics were avoided during hypothermic episodes.

Implementation of this structured plan reduced the frequency and severity of episodes. Strengths of this case include radiologically proven ACC and clear documentation of nadir temperatures (31.3-33.6 °C). Limitations include variability in the documentation of hypothermic episodes.

The analysis is constrained by its single-patient nature; however, detailed chronological documentation allows clinicians to recognize similar presentations. Interpretations are limited to observed data and established literature without implying causality.

## Conclusions

In older adults with unexplained recurrent hypothermia and sweating, clinicians should consider classic Shapiro syndrome, particularly when imaging confirms agenesis of the corpus callosum. A conservative, protocol-driven approach, comprising active rewarming, hydration, medication optimization, and explicit care-home instructions, can markedly reduce morbidity and prevent unnecessary hospital admissions. Through this case report, we hope to encourage further documentation of similar cases and raise awareness of this rare condition.

## References

[1] Shapiro WR, Williams GH, Plum F (1969). Spontaneous recurrent hypothermia accompanying agenesis of the corpus callosum. Brain.

[2] Ren L, Gang X, Yang S, Sun M, Wang G (2022). A new perspective of hypothalamic disease: Shapiro's syndrome. Front Neurol.

[3] Attout H, Amichi S, Belkheir Y (2020). Spontaneous periodic hypothermia in the elderly: a rare or under-recognised syndrome. Eur J Case Rep Intern Med.

[4] Teasdale G, Jennett B (1974). Assessment of coma and impaired consciousness. A practical scale. Lancet.

[5] Pazderska A, O'Connell M, Pender N, Gavin C, Murray B, O'Dowd S (2013). Insights into thermoregulation: a clinico-radiological description of Shapiro syndrome. J Neurol Sci.

[6] Blondin NA (2014). Diagnosis and management of periodic hypothermia. Neurol Clin Pract.

[7] Klingler ET, Meyer K (2004). Shapiro's syndrome: a renewed appreciation for vital signs. Clin Infect Dis.

